# Retraction: Decreased long intergenic noncoding RNA P7 predicts unfavorable prognosis and promotes tumor proliferation via the modulation of the STAT1-MAPK pathway in hepatocellular carcinoma

**DOI:** 10.18632/oncotarget.28366

**Published:** 2024-01-16

**Authors:** Sijie Cheng, Tieling Li, Cheng Wang, Keyu Wang, Chengcai Lai, Jin Yan, Hongxia Fan, Fang Sun, Zhaohai Wang, Peirui Zhang, Linxiang Yu, Zhixian Hong, Guanglin Lei, Baijun Sun, Yuan Gao, Zhaohui Xiao, Xu Ji, Ruilan Wang, Jianzhong Wu, Xiliang Wang, Shaogeng Zhang, Penghui Yang

**Affiliations:** ^1^Beijing 302 Hospital, Beijing, 100039, China; ^2^Chinese PLA General Hospital, Beijing, 100853, China; ^3^State Key Laboratory of Pathogens and Biosecurity, Beijing Institute of Microbiology and Epidemiology, Beijing, 100071, China

**This article has been retracted:** Multiple images in Figure 3C, which illustrates that LincRNA P7 inhibited the proliferation of HCC cells SMMC7721 and Huh7 with colony-forming assay, were found to be fraudulent duplications of images from a previously published Oncotarget paper [1] Figure 2D, which represents results of colony formation in different HCC cells Huh-6, BEL-7402 and SMMC-7721 transfected with unrelated HULC siRNA. As a result, all authors have agreed to the retraction of this paper from Oncotarget.

Original article: Oncotarget. 2018; 9:36057–36066. 36057-36066. https://doi.org/10.18632/oncotarget.23282
